# Mutational analysis of *ITPR1* in a Taiwanese cohort with cerebellar ataxias

**DOI:** 10.1371/journal.pone.0187503

**Published:** 2017-11-29

**Authors:** Cheng-Tsung Hsiao, Yo-Tsen Liu, Yi-Chu Liao, Ting-Yi Hsu, Yi-Chung Lee, Bing-Wen Soong

**Affiliations:** 1 Division of Neurology, Department of Internal Medicine, Taipei Veterans General Hospital Taoyuan Branch, Taoyuan, Taiwan, Republic of China; 2 Department of Neurology, National Yang-Ming University School of Medicine, Taipei, Taiwan, Republic of China; 3 Graduate Institute of Physiology, College of Medicine, National Taiwan University, Taipei, Taiwan, Republic of China; 4 Department of Neurology, Taipei Veterans General Hospital, Taipei, Taiwan, Republic of China; 5 Brain Research Center, National Yang-Ming University, Taipei, Taiwan, Republic of China; 6 Institute of Neuroscience, National Yang-Ming University, Taipei, Taiwan, Republic of China; Sant Joan de Déu Children′s Hospital, SPAIN

## Abstract

**Background:**

The inositol 1,4,5-triphosphate (IP3) receptor type 1 gene (*ITPR1*) encodes the IP3 receptor type 1 (IP3R1), which modulates intracellular calcium homeostasis and signaling. Mutations in *ITPR1* have been implicated in inherited cerebellar ataxias. The aim of this study was to investigate the role of *ITPR1* mutations, including both large segmental deletion and single nucleotide mutations, in a Han Chinese cohort with inherited cerebellar ataxias in Taiwan.

**Methodology and principal findings:**

Ninety-three unrelated individuals with molecularly unassigned spinocerebellar ataxia selected from 585 pedigrees with autosomal dominant cerebellar ataxias, were recruited into the study with elaborate clinical evaluations. The quantitative PCR technique was used to survey large segmental deletion of *ITPR1* and a targeted sequencing approach was applied to sequence all of the 61 exons and the flanking regions of *ITPR1*. A novel *ITPR1* mutation, c.7721T>C (p.V2574A), was identified in a family with dominantly inherited cerebellar ataxia. The proband has an adult-onset non-progressive pure cerebellar ataxia and her daughter is afflicted with a childhood onset cerebellar ataxia with intellectual sub-normalities.

**Conclusion:**

*ITPR1* mutation is an uncommon cause of inherited cerebellar ataxia, accounting for 0.2% (1/585) of patients with dominantly inherited cerebellar ataxias in Taiwan. This study broadens the mutational spectrum of *ITPR1* and also emphasizes the importance of considering *ITPR1* mutations as a potential cause of inherited cerebellar ataxias.

## Introduction

The inositol 1,4,5-triphosphate (IP3) receptor type 1 gene (*ITPR1*) encodes the IP3 receptor type 1 (IP3R1), that functions on the endoplasmic reticulum (ER) membrane in a tetrameric form and modulates intracellular calcium homeostasis and signaling [1]. IP3R1 is abundant in the central nervous system, especially in the Purkinje cells [2, 3]. Mutations in *ITPR1* have been implicated in a small group of patients with cerebellar ataxias and different *ITPR1* mutations may result in similar but still distinguishable phenotypes. Heterozygous large segmental deletions of *ITPR1* were first identified in an Australian and two Belgium families with adult-onset, very slowly progressive, pure cerebellar ataxia that was designated as spinocerebellar ataxia (SCA) type 15 (SCA15; MIM #606658) [4]. Soon, patients with similar *ITPR1* mutations were also identified in Japanese families and Caucasian pedigrees in France and United States [5–10]. Missense mutations in *ITPR1* were later found to cause SCA15 [6, 11, 12] or SCA29 that features a congenital non-progressive cerebellar ataxia (MIM #117360) [13–20]. There is a wide phenotypic diversity linked to *ITPR1* missense mutations. Two *ITPR1* missense mutations (p.N587D and p.S1487D, reference sequence NM_001168272) were recognized as a cause of ataxic cerebral palsy [21], while an *ITPR1* missense mutation (p.I2583N, reference sequence NM_001168272) was recently identified to result in severe pontine and cerebellar hypoplasia [22]. Homozygous, compound heterozygous and heterozygous mutations in *ITPR1* could also cause Gillespie syndrome, which features a non-progressive cerebellar ataxia, aniridia, congenital hypotonia, and intellectual sub-normality (MIM #206700) [23, 24].

The important role played by *ITPR1* in the cerebellum is also demonstrated by the following facts. First, the ataxin-1, ataxin-2 and ataxin-3 are important players in the IP3 pathway and aberrations in the IP3 pathway have been shown in patients with SCA1, SCA2 or SCA3 [25]. Second, carbonic anhydrase 8 (CA8) is capable of inhibiting IP3 binding to IP3R1. CA8 mutations disrupt the formation of synapses of the Purkinje cells and are associated with an autosomal dominant spinocerebellar ataxia plus mental retardation [26]. Furthermore, autoantibody to IP3R1 may induce autoimmune cerebellar ataxia in the humans [27, 28]. All these evidences support the association between *ITPR1* mutations and cerebellar ataxia.

Although *ITPR1* mutations have been identified in a small number of cases with inherited cerebellar ataxia, the relevant research in Han Chinese populations remains limited. The aim of this study was to investigate the role of *ITPR1* mutations, including both large deletion and single nucleotide mutations, in a Han Chinese cohort with inherited cerebellar ataxias in Taiwan.

## Methods and subjects

### Patients and ethics statement

Ninety-three individuals with molecularly unassigned SCA, selected from 585 unrelated patients with dominantly inherited cerebellar ataxias, after excluding SCA1, 2, 3, 6, 7, 8, 10, 12, 17, 31, 35, 36 and dentatorubral pallidoluysian atrophy (DRPLA) [29–31], were enrolled. Clinical evaluations included standard neurological examination, Scale for the Assessment and Rating of Ataxia (SARA) [32, 33], as well as nerve conduction studies and electromyography. Brain magnetic resonance image (MRI) and magnetic resonance spectroscopy (MRS), focusing on the cerebellar hemispheres and vermis, were also performed to assess the cerebellar atrophy and metabolite changes. The protocol for this study was approved by the institutional review board of Taipei Veterans General Hospital and all the participants had given written informed consents.

### Detection of large segmental deletions in *ITPR1*

Genomic DNA was extracted from the white blood cells in the peripheral blood with standard protocols. We looked for large segmental deletions in *ITPR1* utilizing the TaqMan^®^ Copy Number Assays kit on an ABI 7500 Fast Real-Time PCR system (Applied Biosystems, Foster City, CA). Eight TaqMan^®^ detecting probes were selected with reference to those reported previously [34]. All of the detecting probes used in this study are listed in the S1 Table.

### Targeted sequencing and variant calling to detect single nucleotide or oligonucleotide variants

Targeted sequencing covering the coding exons and flanking introns of *ITPR1* (reference sequence: NM_001168272) was carried out by using SeqCap EZ Choice Enrichment Kits (Roche/Nimblegen) according to the manufacturer’s protocol. The enriched DNA library was subjected to massively parallel sequencing using HiSeq2000 platform (Illumina^®^) with 100-bp paired-end reads. The generated FASTQ files were aligned to the GRCh38 reference sequence using Novoalign V.2.07.19 and the PICARD tool MarkDuplicates. Calling was performed using SAMtools V.0.18. The resulting calls were annotated with ANNOVAR. Only variants with the following functional consequences: frameshift, stop gain, stop loss, non-synonymous missense or splice site variants and a global minor allele frequency (MAF) < 0.001 (based on the single nucleotide polymorphism database (dbSNP Build 144, http://www.ncbi.nlm.nih.gov/snp/) were kept. Sanger sequencing was used to further validate variants identified by targeted sequencing.

### *In silico* analysis

To predict the pathogenicity of the *ITPR1* sequence variants, multiple bioinformatics softwares were used, including MutationTaster (http://www.mutationtaster.org) [35], SIFT (http://sift.jcvi.org) [36], and Combined Annotation Dependent Depletion (CADD) (http://cadd.gs.washington.edu) [37]. Amino acid sequences of IP3R1 orthologs from multiple species were aligned using the UniProt Website (http://www.uniprot.org) to investigate whether the variation occurs on a phylogenetically conserved amino acid.

## Results

### General information

For the 93 patients with molecularly unassigned autosomal dominant cerebellar ataxia recruited in this study, the average age at symptom onset was 37.2 ± 18.1 years (range from 1 to 74 years). The common symptoms shared by most patients included gait disturbance (87.1%), dysarthria (52.7%) and appendicular incoordination (50.5%). Tremor was observed in only 19.4% of the subjects, and cognitive impairment in 15.1% of the patients. The flow chart outlining selection of the study cohort and demographics of the study cohort could be found in supplementary S1 Fig and S2 Table.

### Genetic analyses

**Fig 1:**
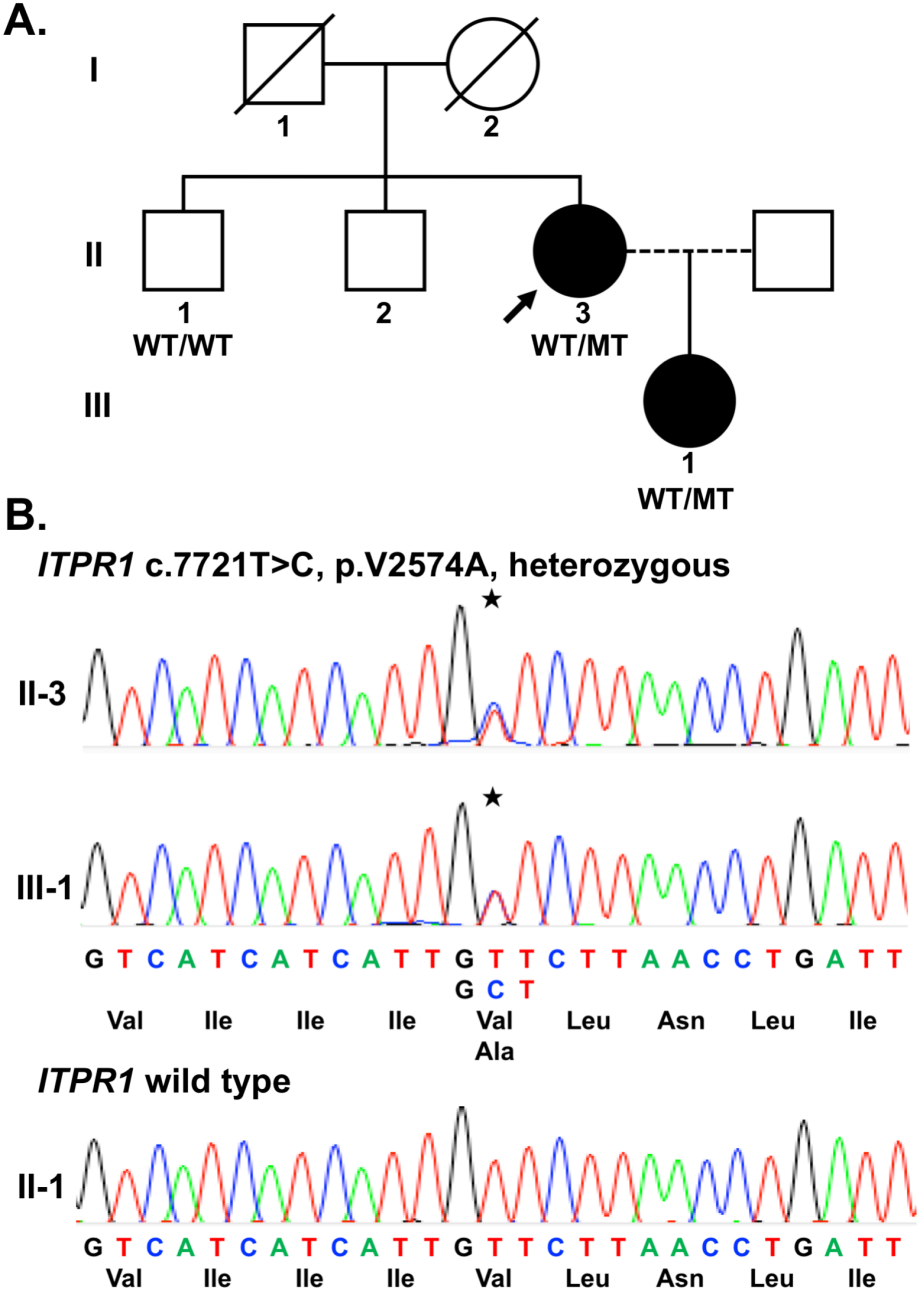
The pedigree and electropherogram of the patients carrying *ITPR1* c.7721T>C mutation. (A) The pedigree of the individuals carrying *ITPR1* p.V2574A (c.7721T>C) mutation in this study. The proband (II-3) is denoted by an arrow. Filled symbols represent symptomatic members, open symbols indicate unaffected individuals, circles stand for females, squares stand for males, WT/WT indicates wild type, and WT/MT stands for individuals harboring the heterozygous mutation. (B) The electropherograms of the patients (II-3 and III-1) carrying the *ITPR1* mutation (WT/MT) and a healthy family member (II-1) carrying two alleles of wild type *ITPR1*. The stars denote the location of the mutation.

We did not identify any individual carrying a large segmental deletion of *ITPR1* (S2 Fig). The targeted sequencing panel, which covered the sequences of all 61 exons and exon-intron boundary regions of *ITPR1*, had a full coverage of the targeted regions and an average depth of 485X for each targeted base. Only one novel missense variant, c.7721T>C (p.V2574A), in exon 58 of *ITPR1* was identified to be a potential causal variant. Sanger sequencing confirmed the variant in the proband (II-3) with dominantly inherited cerebellar ataxia and further revealed that the variant was present in her affected daughter (III-1) and absent in her unaffected brother (II-1) (Fig 1). The variant is not present in 1000 Genomes Project data (1000G, http://www.1000genomes.org/), the single nucleotide polymorphism database (dbSNP Build 144, http://www.ncbi.nlm.nih.gov/snp/) or the Genome Aggregation Database (gnomAD) (http://gnomad.broadinstitute.org).

### Probing the pathogenicity of the *ITPR1* sequence variant c.7721T>C through bioinformatics analyses

**Fig 2:**
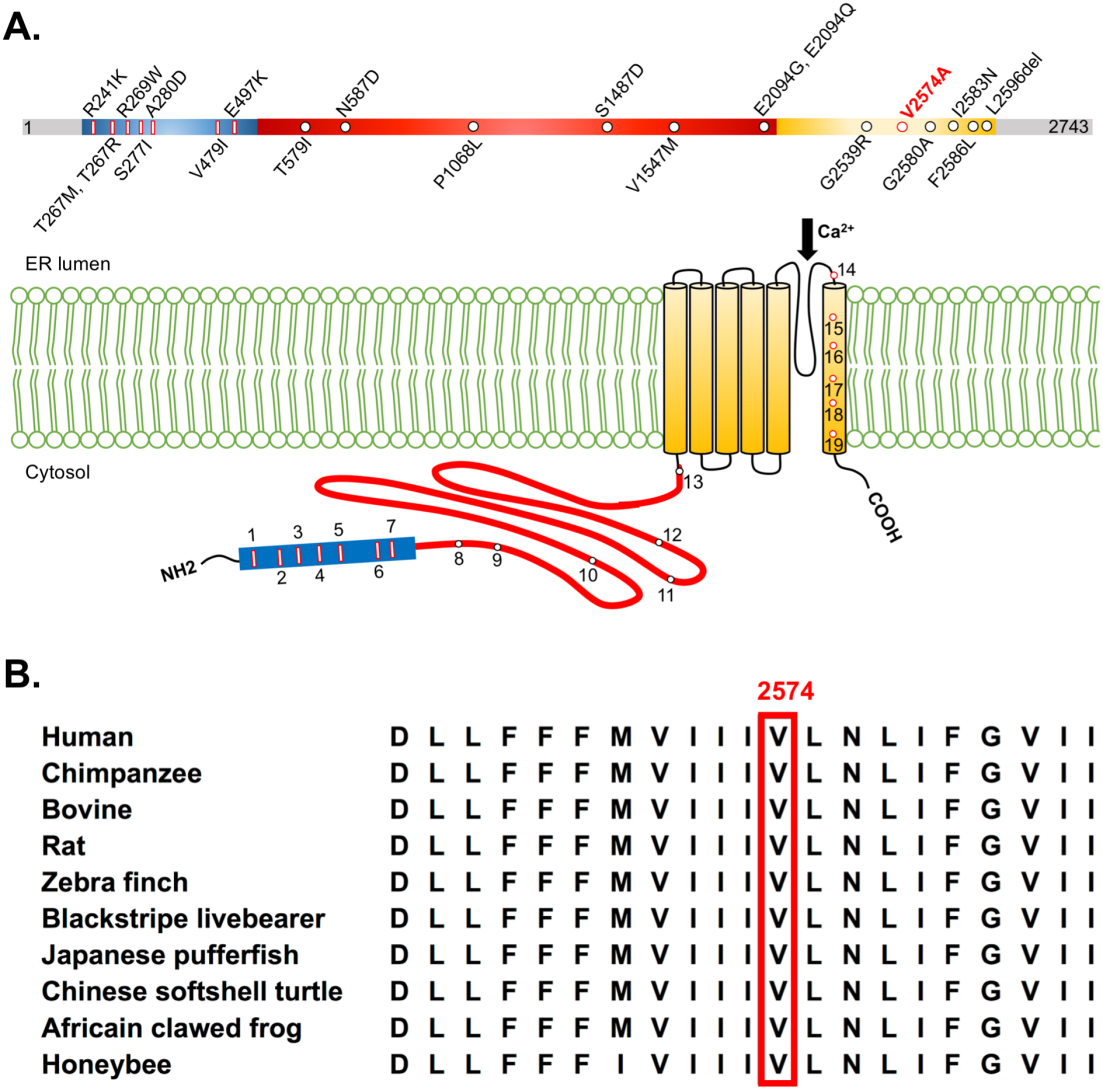
The structure of human IP3R1. (A) The number 1 ~ 19 represent the amino acid residue substitutions reported as far associating with cerebellar ataxia. The blue rectangle represents the IP3 binding region, the red curve line denotes the coupling/regulatory region, and the yellow cylinders stand for the transmembrane segments. (B) The amino acid sequence of the sixth transmembrane segment of human IP3R1 was annotated by the UniProt. The *ITPR1* p.V2574A mutation resides in an evolutionarily conserved region, as shown by aligning the amino acid sequences of IP3R1 protein orthologs from various species. Abbreviation: ER: endoplasmic reticulum ^#^Remarks: All the nucleotide positions and amino acid residues represented here were converted to the reference sequence of NM_001168272 for CDS and NP_001161744 for protein sequence.

*In silico* analysis with MutationTaster predicted this novel variant c.7721T>C (p.V2574A) as disease-causing with a high probability value of 0.999, indicating a high security of this prediction [35]. SIFT predicted c.7721T>C (p.V2574A) as damaging with a score of 0.01 and the threshold of SIFT score for pathogenicity was ≦ 0.05 [36]. The Combined Annotation Dependent Depletion (CADD) program estimated the Phred score of this sequence variant as 27.2, predicting that *ITPR1* p.V2574A was the top 0.19% most deleterious variant in the genome [37]. All of the bioinformatics predictions unanimously supported the pathogenicity of the c.7721T>C (p.V2574A) in *ITPR1* (Table 1). The V2574 residue was highly conserved from human to honeybee, which further supported the pathogenic role of *ITPR1* p.V2574A (Fig 2B). The sequence change was not observed in 1,062 ethnically matched control chromosomes either.

**Table 1:** Bioinformatics analyses of *ITPR1* missense variants.

Mutation	Changes in nucleotide^#^	Changes in amino acid^#^	Prediction program			Reference sequence
			MutationTaster Probability value	SIFT SIFT score	CADD Phred score	
Novel missense in S6 reported in this study	c.7721T>C	p.V2574A	Disease causing 0.999999999677562	Damaging 0.01	27.2	NM_001168672
Reported S6 mutation	c.7739G>C	p.G2580A	Disease causing 0.999999999992046	Damaging 0	27.7	NM_001168672
Reported S6 mutation	c.7748T>A	p.I2583N	Disease causing 0.999999999953971	Damaging 0	33	NM_001168672
Reported mutation with functional analysis	c.3203C>T	p.P1068L	Disease causing 0.999999999999659	Damaging 0.03	32	NM_001168672

^#^Remarks: All the nucleotide positions and amino acid residues represented here have been converted to the reference sequence of NM_001168272 for CDS and NP_001161744 for protein sequence.

Abbreviations: CADD: Combined Annotation Dependent Depletion; S6: the sixth segment of transmembrane domain.

### Clinical characteristics of patients with the *ITPR1* mutation

**Fig 3:**
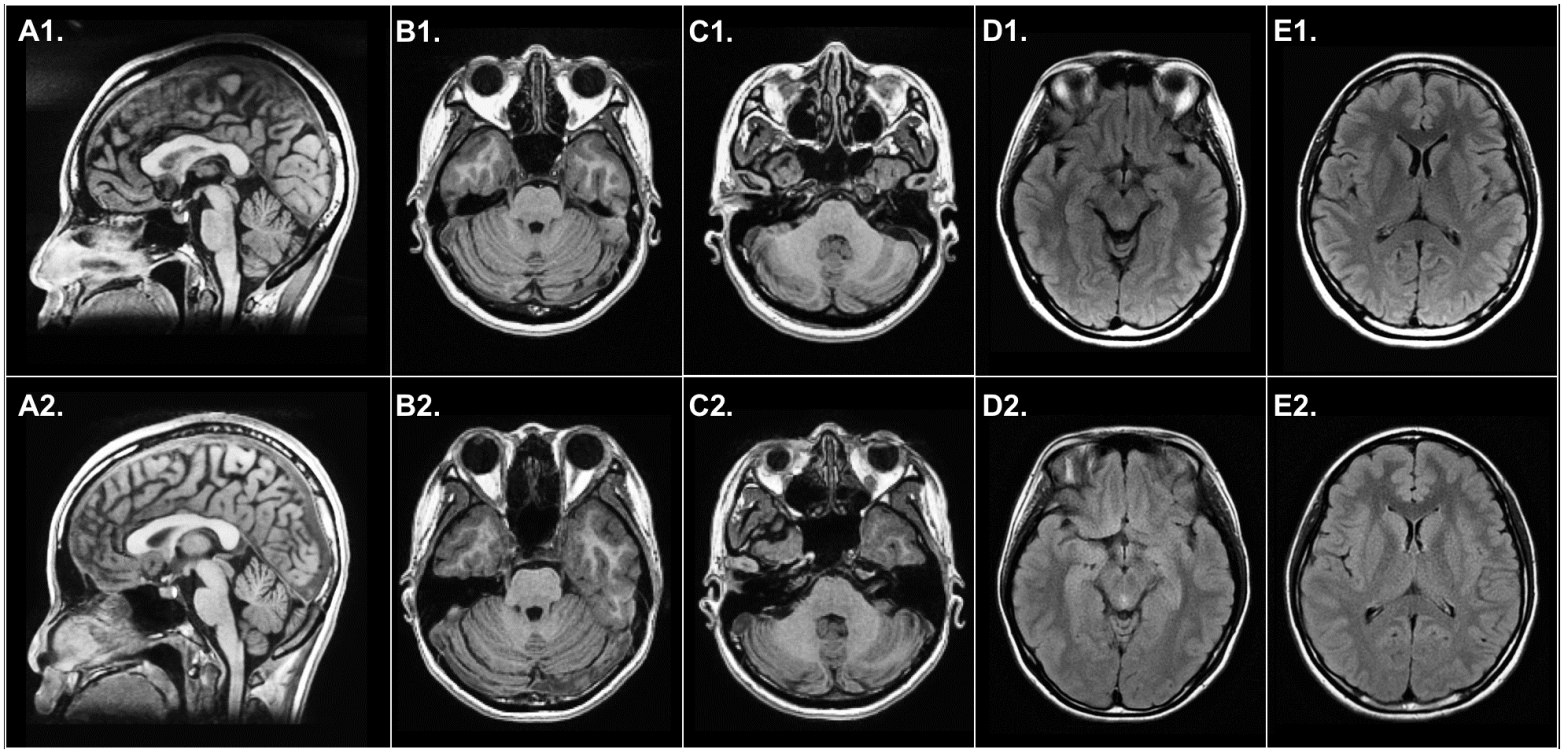
Brain MRI of the patients carrying *ITPR1* mutation. The neuroimages of II-3 are shown as A1-E1, and her daughter’s images (III-1) are A2-E2. The T1-weighted sagittal view images denote a mild atrophy of the anterior and posterior lobes of the cerebellar vermis (A1 and A2). The T1-weighted axial view images demonstrate a mild atrophy of the cerebellar hemispheres (B1-C1 and B2-C2). The sizes of the pons and cerebellar peduncles are within normal ranges. The fluid-attenuated inversion recovery (FLAIR) axial view image features normal cerebral cortex, basal ganglia and midbrain (D1-E1 and D2-E2).

The pedigree of our SCA patients with *ITPR1* p.V2574A mutation is shown in Fig 1A. The clinical characteristics of the index case and her offspring were summarized in Table 2. The proband (II-3, Fig 1A) had experienced an insidious and non-progressive gait disturbance since age 30. Upon examination at age 35 years, she was found to have fine tremors over the head and hands, especially when getting nervous, and her hand-writing was sloppy. Mild dysarthria and dysphagia were also noticed. She had normal cognitive functions. The eye movements were full in all directions but with mild saccadic pursuits. There was no focal weakness and the muscle tone was normal. Diffusely decreased deep tendon reflexes were found with flexor plantar responses. There was mild unsteadiness on tandem walking. The latest SARA score was 4.5 at the tenth year after the disease onset (S1 Video). Nerve conduction studies and electromyography were unremarkable. Brain MRI at the tenth year after disease onset demonstrated a few tiny non-specific hyperintense spots in the white matter of cerebral hemispheres on T2-weighted images and a mild atrophy of the cerebellar hemispheres and vermis on T1-weighted images (Fig 3). The MRS revealed NAA/Cr ratios of 1.16, 1.02 and 0.87 in the right cerebellar hemisphere, left cerebellar hemisphere and cerebellar vermis, respectively, indicating a very mild biochemical abnormality.

**Table 2:** The clinical characteristics of the cases reported in this paper.

Mutation	Sex	Age at onset	First symptom	Clinical course	Symptoms	SARA scores	MRI findings
c.7721T>C, p.V2574A	Female (II-3)	30	Unsteady gait	Non-progressive	Dysarthria, dysphagia, mild saccadic pursuits, sloppy hand writing, fine tremors of the head and hands	0–3.5–1–1–1.5 at a 6-month interval at age 32–35; 4.5 at the 10^th^ year after the onset	Mild atrophy of the vermis and cerebellar hemispheres
	Female (III-1)	7	Unsteady gait	Non-progressive	Cognitive sub-normalities (MMSE: 25), mild dysarthria, saccadic pursuits, sloppy hand writing, easy falling	5.5 at the 13^th^ year after the onset	Mild atrophy of the vermis and cerebellar hemispheres

Abbreviations: MMSE: Mini-Mental State Examination; SARA: Scale for the Assessment and Rating of Ataxia.

The proband’s daughter (III-1) had experienced a childhood-onset non-progressive cerebellar ataxia and cognitive impairment since age of 7 years. Mild slurred speech, saccadic pursuits and unsteady tandem walking on neurological examination, a SARA score of 5.5 (S2 Video) and mini-mental state examination (MMSE) score of 25 were documented at the 13^th^ year after the symptom onset. The nerve conduction studies were normal. Brain MRI featured a mild atrophy of the cerebellar hemispheres and vermis on the T1-weighted images (Fig 3). There was no obvious biochemical abnormality in the right cerebellar hemisphere, left cerebellar hemisphere or vermis (NAA/Cr ration of 1.55, 1.25 and 1.02, respectively) on MRS at the 13^th^ year after the onset.

## Discussion

We identified a novel missense *ITPR1* mutation, c.7721T>C (p.V2574A), in a patient with a slowly progressive cerebellar ataxia from 93 unrelated patients with molecularly unassigned SCA. Since these 93 patients were selected from a cohort of 585 unrelated patients with dominantly inherited cerebellar ataxias after a comprehensive genetic testing for SCA, mutation in *ITPR1* accounts for 0.2% (1/585) of dominantly inherited cerebellar ataxias in Taiwan. Several lines of evidence support the pathogenicity of this *ITPR1* mutation, namely, p.V2574A. First, the sequence change was absent in 1,062 ethnically matched control chromosomes. Second, the variant was absent in several genetic polymorphism databases, including dbSNP, 1000 Genomes Project and gnomAD database. Third, the variant occurs at an evolutionarily conserved amino acid residue of the IP3R1 protein (Fig 2) and a deleterious effect was unanimously predicted by several bioinformatics programs, including MutationTaster [35], SIFT [36] and CADD [37] (Table 1). Furthermore, this variant has a perfect genotype-phenotype co-segregation in the pedigree (Fig 1). These evidences substantially support the pathogenicity of this mutation and classify it as a “likely pathogenic variant” according to the guideline for the interpretation of sequence variants recommended by the American College of Medical Genetics and Genomics and the Association of Molecular Pathology [38].

So far, there have been 16 mutations in *ITPR1* associated with autosomal dominant cerebellar ataxia and 5 with dominantly inherited Gillespie syndrome (Table 3 and Fig 2A). Most of the mutations locate in the IP3 binding domain and the coupling/regulatory region of IP3R1. Mutations localized in the coupling/regulatory domain of IP3R1, such as *ITPR1* p.P1068L (originally annotated as p.P1059L, reference sequence NM_002222 [6]), might affect the binding affinity and transmission of the IP3 signaling which further disrupts the calcium influx, resulting in cerebellar ataxia [11]. Similar to *ITPR1* p.G2580A (annotated as p.G2547A, reference sequence NM_001099952, in the original literature [17]) and p.I2583N (annotated as p.I2550N, reference sequence NM_001099952, in the original literature [22]) mutations, the p.V2574A mutation identified in this study is also situated in the sixth transmembrane domain (S6) adjacent to the S5-S6 pore-loop. As a result, these three mutations might alter the structure of the calcium-selective channel and affect the calcium influx through the channel. Moreover, since the functional IP3R1 is a tetrameric structure [39,40], mutations in the transmembrane domain might disrupt the assembly conformation of the functional channels which is essential for the intracellular calcium release (Fig 2 and S3 Fig).

**Table 3:** The molecular and clinical characteristics of *ITPR1*-associated autosomal dominant cerebellar ataxias in the literature.

Reference ^#1^	Methods	Clinical presentation	Diagnosis	Mutation^#2^	Sites^#3^	Domain
Barresi S, 2016 [19]	TS and WES	Congenital non-progressive CA	Not available	c.722G>A, p.R241K	1	IP3-binding domain
				c.805C>T, p.R269W	3	IP3-binding domain
				c.839C>A, p.A280D	5	IP3-binding domain
				c.1489G>A, p.E497K	7	IP3-binding domain
Sasaki M, 2015 [18]	WES	Childhood-onset non-progressive CA, sporadic, motor developmental delay and mild cognitive deficits, nystagmus, tremor	SCA29	c.800C>G, p.T267R	2	IP3-binding domain
				c.830G>T, p.S277I	4	IP3-binding domain
				c.1736C>T, p.T579I	8	Coupling/regulatory region
Ohba C, 2013 [15]	WES	Congenital CA, hypotonia, nystagmus, slurred speech, motor development delay	SCA29	c.800C>T, p.T267M	2	IP3-binding domain
Ganesamoorthy D, 2009 [12]	MLPA	Idiopathic ataxia	SCA15	c.1435G>A, p.V479I	6	IP3-binding domain
Parolin Schnekenberg R, 2015 [21]	WES	Congenital CA, nystagmus, delayed walking, intellectual disability	Ataxic CP	c.1759A>G, p.N587D	9	Coupling/regulatory region
				c.4459_4460DelinsGA, p.S1487D	11	Coupling/regulatory region
Huang L, 2012 [14]	WES	Delayed sitting until age of 8 months, gaze-evoked nystagmus, hypotonia, titubation, fine motor deficits, delayed language learning, seizure at age of 5 years	SCA29	c.1759A>G, p.N587D	9	Coupling/regulatory region
		Poor balance and coordination since a few months of age, ataxic gait, mild developmental delay	SCA29	c.4639G>A, p.V1547M	12	Coupling/regulatory region
Hara K, 2008 [6], Yamazaki H, 2011 [11]	Mutational analysis	Age of onset between 12 and 35 years, truncal and limbs ataxia, with alternative tremor or myoclonus, atrophy of cerebellar vermis and hemispheres	SCA15	c.3203C>T, p.P1068L	10	Coupling/regulatory region
Shadrina MI, 2016 [20]	WES	AD, non-progressive ataxia, mild symptoms, normal cognition	SCA29	c.4639G>A, p.V1547M	12	Coupling/regulatory region
McEntagart M, 2016 [24]	WES and TS	Congenital iris hypoplasia, cerebellar ataxia, hypotonia, and intellectual impairment	Gillespie syndrome, AD	c.6280G>C, p.E2094Q	13	Coupling/regulatory region
				c.6281A>G, p.E2094G	13	Coupling/regulatory region
				c.7615G>A, p.G2539R	14	Transmembrane domain segment 5–6
				c.7786_ 7788delAAG, p.L2596del	19	Transmembrane domain segment 6
The present study	qPCR and TS	The proband features an adult-onset non-progressive cerebellar ataxia with tremor; The offspring features childhood-onset non-progressive cerebellar ataxia with intellectual sub-normalities	Non-progressive cerebellar ataxi	c.7721T>C, p.V2574A	15	Transmembrane domain segment 6
Gonzaga-Jauregui C, 2015 [17]	WES	Neuropathy and congenital non-progressive cerebellar ataxia	SCA29	c.7739G>C, p.G2580A	16	Transmembrane domain segment 6
van Dijk T, 2017 [22]	WES	Delayed motor development at age of 6 months, ataxia, cognitive sub-normality	PCH with ataxia	c.7748T>A, p.I2583N	17	Transmembrane domain segment 6
Gerber S, 2016 [23]	WES	Congenital iris hypoplasia, cerebellar ataxia, generalized hypotonia, with/without intellectual impairment	Gillespie syndrome, AD	c.7758T>G, F2586L	18	Transmembrane domain segment 6
				c.7786_ 7788delAAG, p.L2596del	19	Transmembrane domain segment 6

^#^Remarks:
The references were represented as the first author’s name, year of publication. All the nucleotide position and amino acid residues represented here have been converted to the reference sequence of NM_001168272 for coding sequence (CDS) and NP_001161744 for protein sequence. These sites refer to the number assigned for the amino acid residue on Fig 2. To be noted, some mutations in *ITPR1* were associated with “recessively” inherited Gillespie syndrome [23] and therefore are not listed in Fig 2 and Table 3.

Abbreviations:

AD: autosomal dominant; Ataxic CP: ataxic cerebral palsy; Congenital CA: congenital cerebellar ataxia; MLPA: Multiplex ligation-dependent probe amplification; PCH: pontocerebellar hypoplasia; SCA: spinocerebellar ataxia; *SUMF1*: Sulfatase Modifying Factor 1 gene; TS: targeted sequencing; WES: whole exome sequencing.

The clinical features of the individuals with *ITPR1*-associated cerebellar ataxia are quite heterogeneous. Tremors and vermis atrophy are common but not pathognomonic. The onset of ataxia could be in the infantile stage, early childhood [14, 15, 17–19, 21], or adulthood [6, 20], and intellectual impairment were frequently found in the individuals with an earlier age at onset of disease. Our pedigree demonstrated a certain diversity in the clinical presentations. The proband (II-3) presented with an adult-onset ataxia but her offspring (III-1) featured a childhood-onset ataxia and intellectual sub-normalities. Both cases demonstrated non-progressive cerebellar ataxia and mild atrophy of the vermis. These inter-familial and intra-familial heterogeneities suggest that there might be modifying factors underlying the pathogenesis of *ITPR1*-associated cerebellar ataxia.

There are several validated methods to detect copy number variation (CNV) in the genome, including multiplex ligation-dependent probe amplification (MLPA) [12, 41], array comparative genomic hybridization (CGH) [6], qPCR [9, 10, 34] and fine sequence array. We first looked for CNV utilizing the qPCR method with multiple probes covering most of the deletion hot spots but failed to find any *ITPR1* large deletions in our cohort. We further surveyed *ITPR1* mutations with high throughput targeted sequencing and found an *ITPR1* missense mutation. Our findings emphasize the importance of using multiple diagnostic tools to detect both CNV and single nucleotide variants in *ITPR1*. *ITPR1* missense mutations-associated cerebellar ataxia might have been under-estimated prior to the high-throughput sequencing era. Given that IP3R1 consists of 2,743 amino acids and the full-length *ITPR1* gene is composed of 10,197 base pairs and 61 exons, it is technically challenging and time- and cost-consuming to perform genetic screening in such a large gene. The diagnostic proficiency has significantly improved with the next generation sequencing technology. Researches focusing on the missense mutation in *ITPR1* might be much feasible from now on.

*ITPR1* missense mutation is an uncommon but unneglectable cause of cerebellar ataxia in Taiwan. Cases with non-progressive cerebellar ataxia and vermis atrophy might be candidates with mutations in *ITPR1*. Our findings have broadened the mutational spectrum of *ITPR1* and emphasized the role of *ITPR1* in cerebellar function.

## Supporting information

S1 Table Probes used in qPCR for detecting copy number variation. (DOCX)

S2 Table Demographics of the study cohort. (DOCX)

S1 Fig Flow chart outlining selection of the study cohort. (TIFF)

S2 Fig Average estimated copy number detected by each probes used in the copy number analysis with qPCR technique. (TIFF)

S3 Fig The crystallographic structure of IP3R1. (TIFF)

S1 Video SARA assessment of patient II-3. (MP4)

S2 Video SARA assessment of patient III-1. (MP4)
