# Correction: Mutational analysis of *ITPR1* in a Taiwanese cohort with cerebellar ataxias

**DOI:** 10.1371/journal.pone.0192866

**Published:** 2018-02-08

**Authors:** Cheng-Tsung Hsiao, Yo-Tsen Liu, Yi-Chu Liao, Ting-Yi Hsu, Yi-Chung Lee, Bing-Wen Soong

The legends for the Supporting Information files S2 Fig and S3 Fig are missing. Please see the complete captions below.

## Supporting information

S2 Fig Average estimated copy number detected by each probes used in the copy number analysis with qPCR technique. The black and gray bars indicate individuals with cerebellar ataxia and neurologically healthy controls, respectively, recruited in this study. The average estimated copy number for each probes was around 2, similar to those of the healthy controls.
(TIFF)

S3 Fig The crystallographic structure of IP3R1. The sequences between the human and rat IP3R1 are 98.5% identical. The crystallographic structure of rat IP3R1 (PDB: 3JAV) [39] was visualized with UCSF Chimera software [40]. Each single subunit of IP3R1 was colored with yellow, green, pink and blue, respectively. The residue V2574 was labeled with black. V2574 residue locating near the pore-forming region was visualized by the side-view of a single subunit of IP3R1 (A), the side-view of a tetrameric IP3R1 structure (B), the top-view of a tetrameric IP3R1 structure (C), and a close-up top-view focusing on the V2574 residue of each subunit (D).
(TIFF)
