# Supplementary material for: Mutational analysis of ITPR1 in a Taiwanese cohort with cerebellar ataxias
Source: PLoS One. 2017 Nov 29;12(11):e0187503. doi: 10.1371/journal.pone.0187503 (PMC5706750; doi:10.1371/journal.pone.0187503)
Supplement: S1 Table — (DOCX) [file pone.0187503.s001.docx]

**S1 Table. Probes used in qPCR for detecting copy number variation.**

| *ITPR1*_NCBI location：[Chromosome 3: 4,535,032-4,889,524](http://grch37.ensembl.org/Homo_sapiens/Location/View?db=core;g=ENSG00000150995;r=3:4535032-4889524;t=ENST00000302640) | | | | | |  |
| --- | --- | --- | --- | --- | --- | --- |
| Distance | Location | Exon/Intron location | Probe | Assay Location | NCBI location | Reported |
| 0 | 4536174 | Exon 2 | Hs02086003_cn | Overlaps Exon 2 - Intron 2 | Chr.3:4536174 on NCBI build 37 | Yes [34] |
| 129140 | 4665314 | Intron 4 | Hs06700310_cn | Intron 4 | Chr.3:4665314 on NCBI build 37 | Yes [34] |
| 22066 | 4687380 | Exon 10 | Hs02622322_cn | Overlaps Exon 10 - Intron 10 | Chr.3:4687380 on NCBI build 37 |  |
| 25119 | 4712499 | Exon 20 | Hs01962250_cn | Exon 20 | Chr.3:4712499 on NCBI build 37 |  |
| 35474 | 4747973 | Exon 37 | Hs02055462_cn | Exon 37 | Chr.3:4747973 on NCBI build 37 |  |
| 11663 | 4759636 | Intron 40 | Hs04721273_cn | Intron 40 | Chr.3:4759636 on NCBI build 37 |  |
| 59324 | 4818960 | Exon 48 | Hs00458805_cn | Overlaps Intron 47 - Exon 48 | Chr.3:4818960 on NCBI build 37 | Yes [34] |
| 37936 | 4856896 | Exon 59 | Hs02591037_cn | Overlaps Exon 59 - Intron 59 | Chr.3:4856896 on NCBI build 37 | Yes [34] |
