# Supplementary material for: Mutational analysis of ITPR1 in a Taiwanese cohort with cerebellar ataxias
Source: PLoS One. 2017 Nov 29;12(11):e0187503. doi: 10.1371/journal.pone.0187503 (PMC5706750; doi:10.1371/journal.pone.0187503)
Supplement: S2 Table — (DOCX) [file pone.0187503.s002.docx]

**S2 Table: Demographics of the study cohort.**

| Numbers of patients (M/F) | 93 (37/56) |
| --- | --- |
| Age at onset (mean ± SD, years) | 37.2 ± 18.1 |
| Frequency of symptoms (%) |  |
| Gait disturbance | 87.1 |
| Dizziness | 20.4 |
| Dysarthria | 52.7 |
| Dysphagia | 12.9 |
| Appendicular incoordination | 50.5 |
| Eye movement aberrations | 31.2 |
| Nystagmus | 23.7 |
| Hearing impairment | 3.2 |
| Visual impairment | 5.4 |
| Cognitive impairment | 15.1 |
| Tremor | 19.4 |

Abbreviations: M: male; F: female; SD: standard deviation.
