# Supplementary figures and images for: Mutational analysis of ITPR1 in a Taiwanese cohort with cerebellar ataxias
Source: PLoS One. 2017 Nov 29;12(11):e0187503. doi: 10.1371/journal.pone.0187503 (PMC5706750; doi:10.1371/journal.pone.0187503)

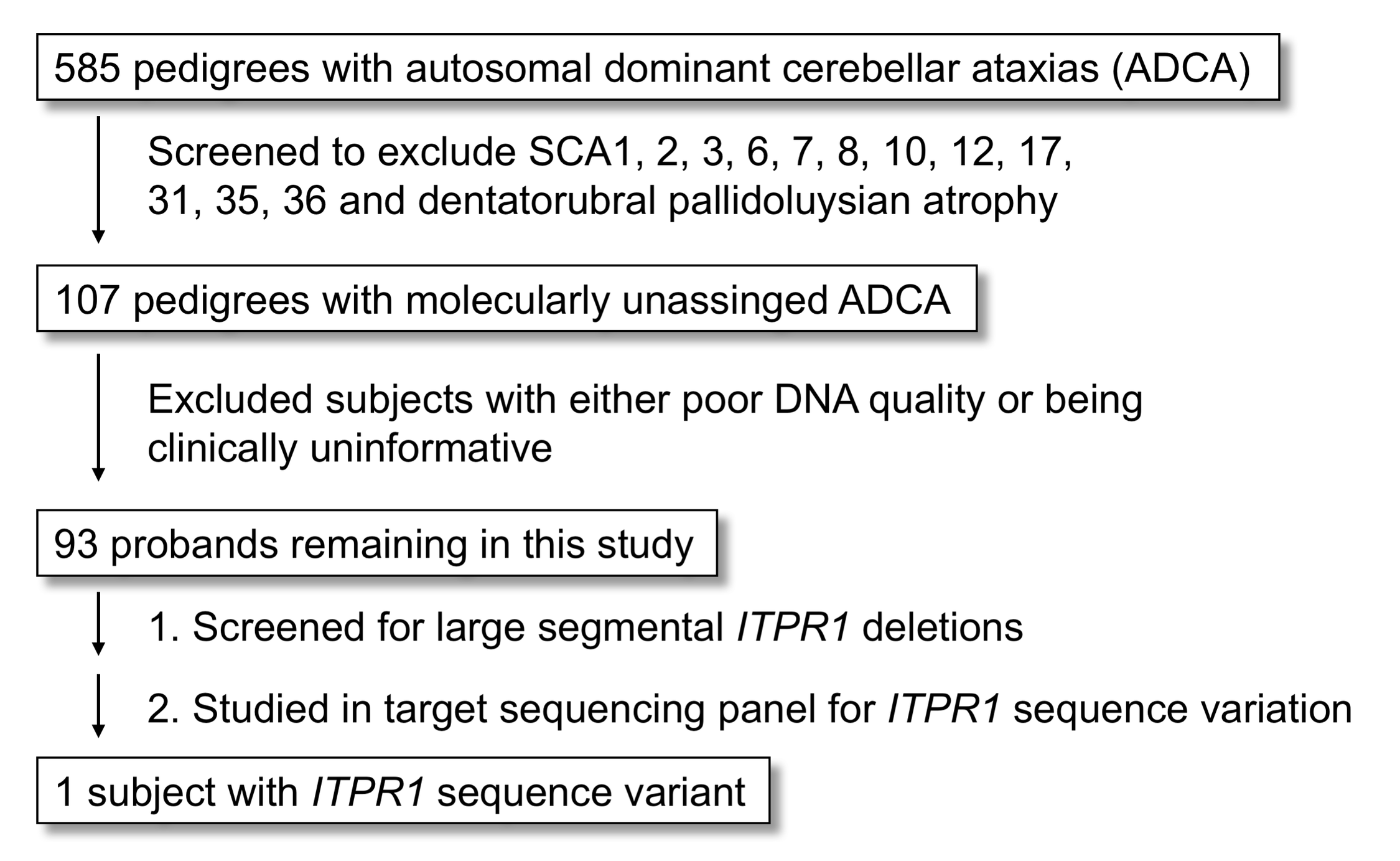

Supplement: S1 Fig — (TIFF) [file pone.0187503.s003.tiff]

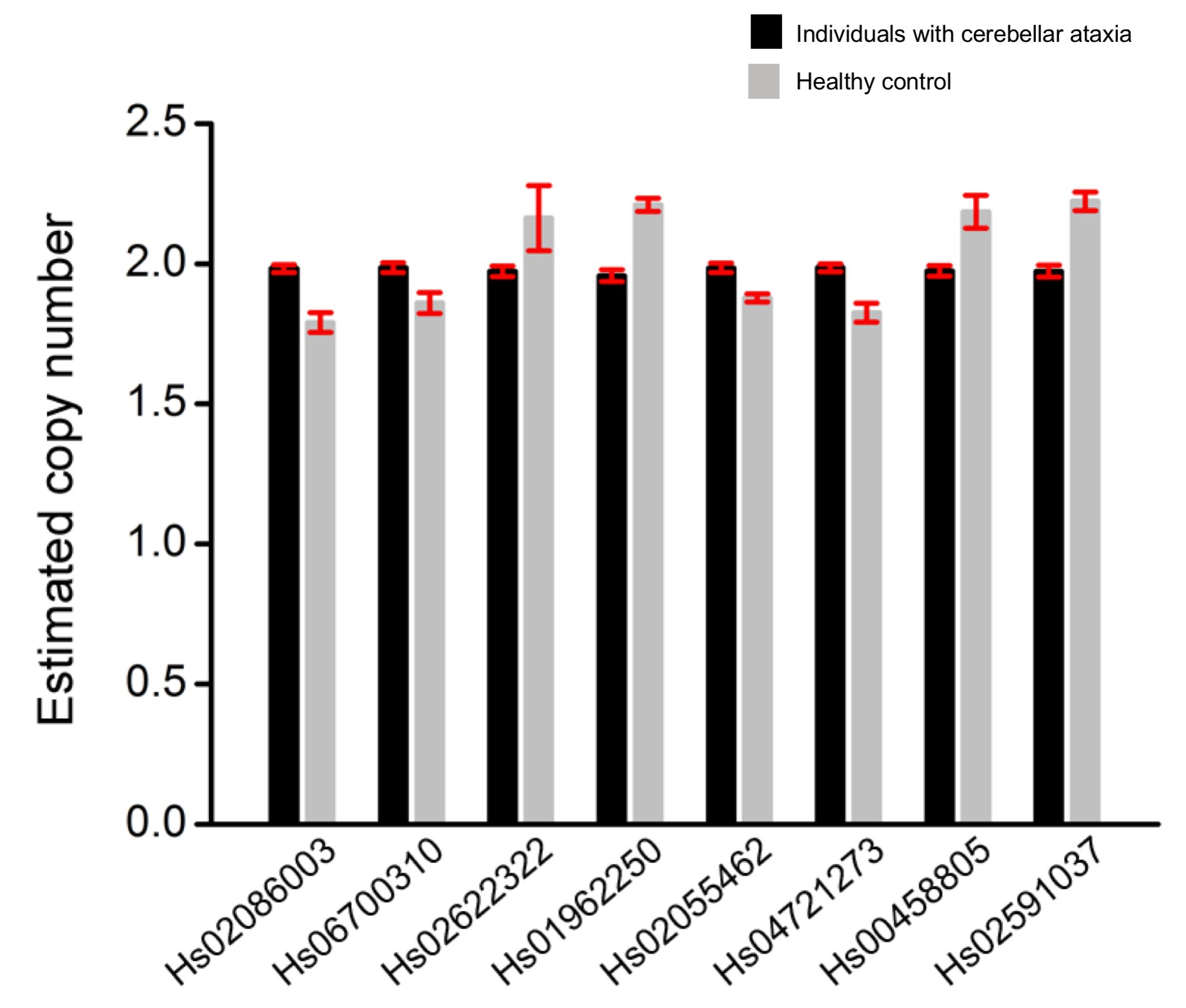

Supplement: S2 Fig — (TIFF) [file pone.0187503.s004.tiff]

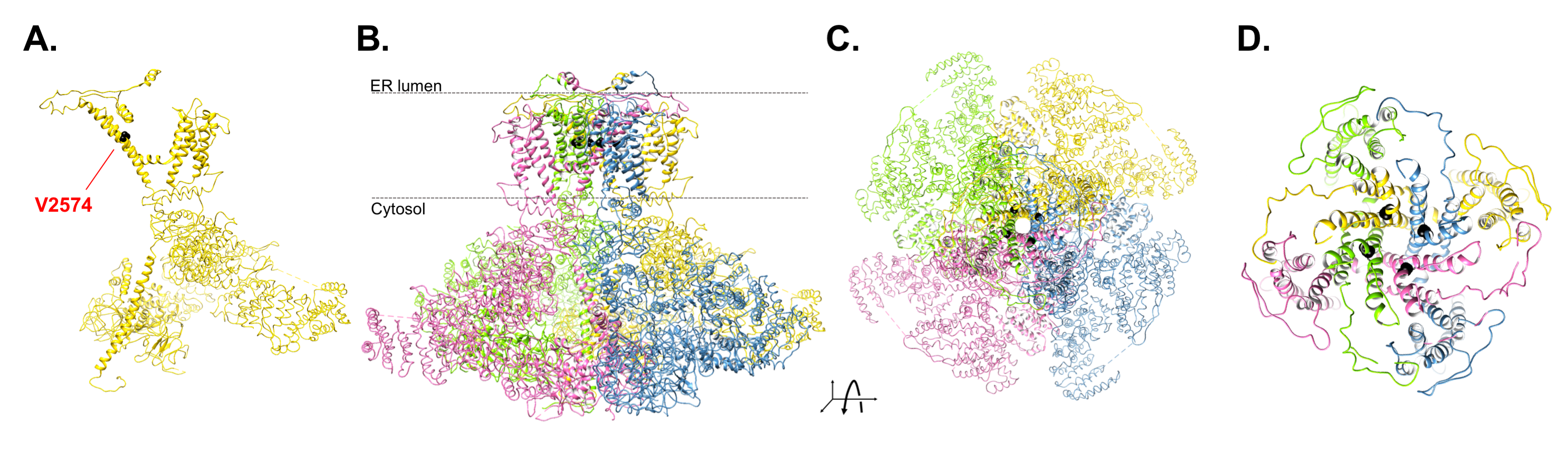

Supplement: S3 Fig — (TIFF) [file pone.0187503.s005.tiff]
