# Supplementary figures and images for: Correction: Mutational analysis of ITPR1 in a Taiwanese cohort with cerebellar ataxias
Source: PLoS One. 2018 Feb 8;13(2):e0192866. doi: 10.1371/journal.pone.0192866 (PMC5805335; doi:10.1371/journal.pone.0192866)

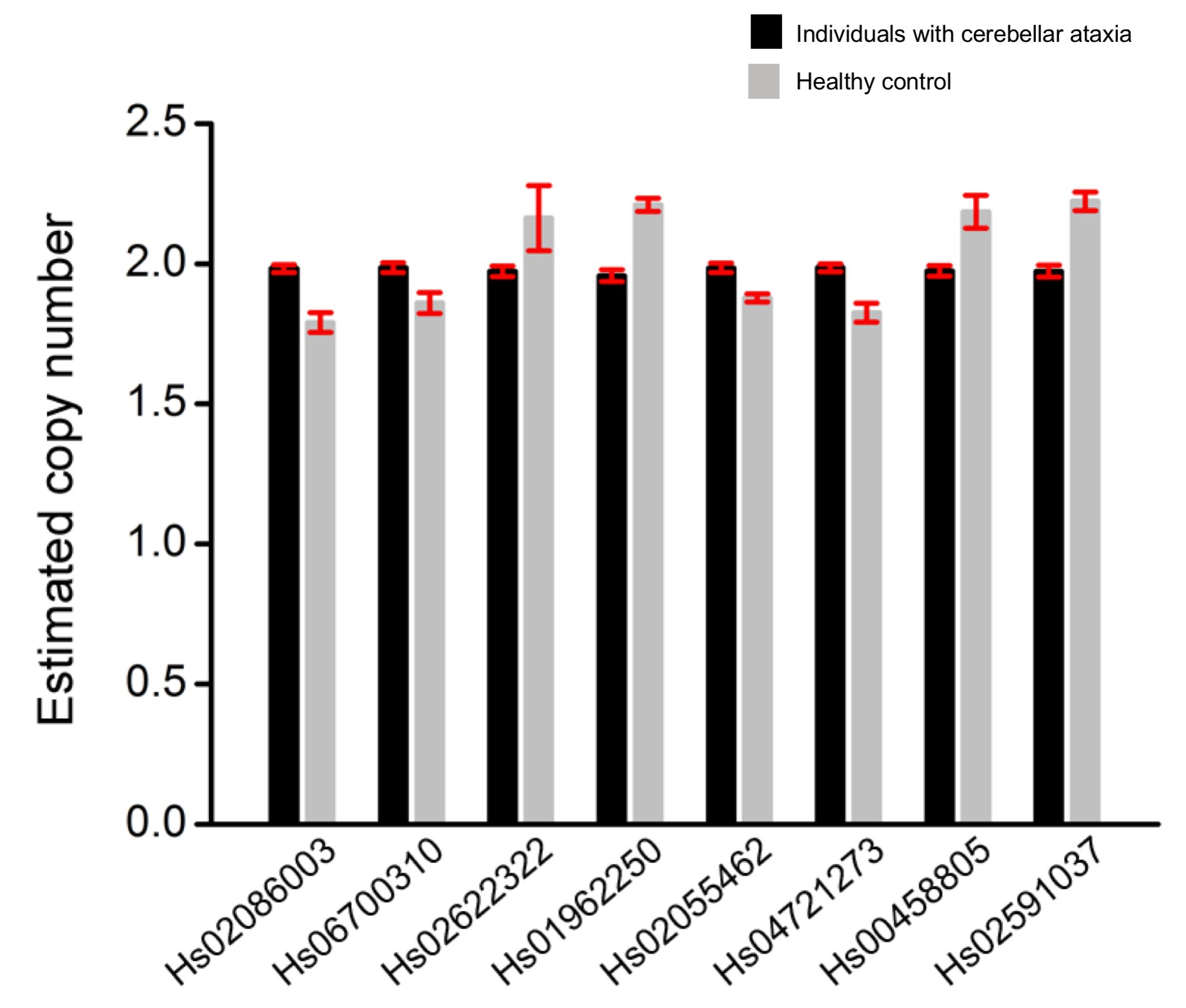

Supplement: S2 Fig — The black and gray bars indicate individuals with cerebellar ataxia and neurologically healthy controls, respectively, recruited in this study. The average estimated copy number for each probes was around 2, similar to those of the healthy controls. (TIFF) [file pone.0192866.s001.tiff]

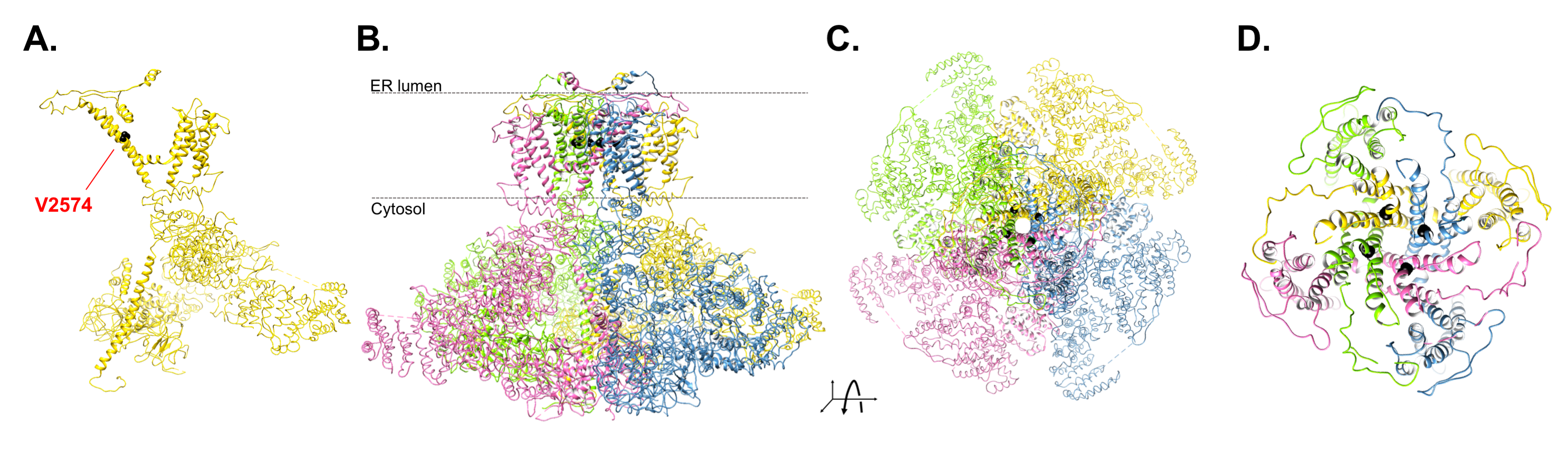

Supplement: S3 Fig — The sequences between the human and rat IP3R1 are 98.5% identical. The crystallographic structure of rat IP3R1 (PDB: 3JAV) [39] was visualized with UCSF Chimera software [40]. Each single subunit of IP3R1 was colored with yellow, green, pink and blue, respectively. The residue V2574 was labeled with black. V2574 residue locating near the pore-forming region was visualized by the side-view of a single subunit of IP3R1 (A), the side-view of a tetrameric IP3R1 structure (B), the top-view of a tetrameric IP3R1 structure (C), and a close-up top-view focusing on the V2574 residue of each subunit (D). (TIFF) [file pone.0192866.s002.tiff]
